# The Role of Social Interactions in Motor Performance: Feasibility Study Toward Enhanced Motivation in Telerehabilitation

**DOI:** 10.2196/12708

**Published:** 2019-05-15

**Authors:** Roni Barak Ventura, Shinnosuke Nakayama, Preeti Raghavan, Oded Nov, Maurizio Porfiri

**Affiliations:** 1 Department of Mechanical and Aerospace Engineering New York University Tandon School of Engineering Brooklyn, NY United States; 2 Department of Rehabilitation Medicine New York University School of Medicine New York, NY United States; 3 Department of Technology Management and Innovation New York University Tandon School of Engineering Brooklyn, NY United States; 4 Department of Biomedical Engineering New York University Tandon School of Engineering Brooklyn, NY United States

**Keywords:** citizen science, social interactions, telerehabilitation, physical therapy

## Abstract

**Background:**

Robot-mediated telerehabilitation has the potential to provide patient-tailored cost-effective rehabilitation. However, compliance with therapy can be a problem that undermines the prospective advantages of telerehabilitation technologies. Lack of motivation has been identified as a major factor that hampers compliance. Exploring various motivational interventions, the integration of citizen science activities in robotics-based rehabilitation has been shown to increase patients’ motivation to engage in otherwise tedious exercises by tapping into a vast array of intrinsic motivational drivers. Patient engagement can be further enhanced by the incorporation of social interactions.

**Objective:**

Herein, we explored the possibility of bolstering engagement in physical therapy by leveraging cooperation among users in an environmental citizen science project. Specifically, we studied how the integration of cooperation into citizen science influences user engagement, enjoyment, and motor performance. Furthermore, we investigated how the degree of interdependence among users, such that is imposed through independent or joint termination (JT), affects participation in citizen science-based telerehabilitation.

**Methods:**

We developed a Web-based citizen science platform in which users work in pairs to classify images collected by an aquatic robot in a polluted water canal. The classification was carried out by labeling objects that appear in the images and trashing irrelevant labels. The system was interfaced by a haptic device for fine motor rehabilitation. We recruited 120 healthy volunteers to operate the platform. Of these volunteers, 98 were cooperating in pairs, with 1 user tagging images and the other trashing labels. The other 22 volunteers performed both tasks alone. To vary the degree of interdependence within cooperation, we implemented independent and JTs.

**Results:**

We found that users’ engagement and motor performance are modulated by their assigned task and the degree of interdependence. Motor performance increased when users were subjected to independent termination (*P*=.02), yet enjoyment decreased when users were subjected to JT (*P*=.005). A significant interaction between the type of termination and the task was found to influence productivity (*P*<.001) as well as mean speed, peak speed, and path length of the controller (*P*=.01, *P*=.006, and *P*<.001, respectively).

**Conclusions:**

Depending on the type of termination, cooperation was not always positively associated with engagement, enjoyment, and motor performance. Therefore, enhancing user engagement, satisfaction, and motor performance through cooperative citizen science tasks relies on both the degree of interdependence among users and the perceived nature of the task. Cooperative citizen science may enhance motivation in robotics-based telerehabilitation, if designed attentively.

## Introduction

### Background

Debilitating neurological diseases such as stroke require intensive, repetitive, and high-frequency physical therapy for maximum recovery of motor function and self-reliance [1-3]. However, costly resources and limited rehabilitation personnel make rehabilitation unavailable to the majority of patients. Furthermore, disability often encumbers mobility, preventing patients from leaving their homes and frequenting the therapists’ office [4]. Therefore, reaching their full recovery potential is greatly contingent upon performing self-directed physical therapy with limited professional feedback.

### Robot-Mediated Telerehabilitation

Several rehabilitation robots have been developed for delivery of exercise programs for the upper limb, including the MIT-Manus (Massachusetts Institute of Technology) [5,6], Gentle/S (University of Reading) [7], ArmIn (ETH Zurich) [8], and Mirror Image Movement Enabler (Stanford University) [9]. Ultimately, robotic rehabilitation devices aim to administer and monitor exercise for the arm with reproducible high-intensity and high-dosage sensorimotor therapy while collecting pertinent data for assessment by a medical professional [10,11].

The major hurdles in the widespread adoption of these robotics-based technologies are costs and user-friendliness, whereby these devices often have prohibitive costs to the general public and require some form of technological proficiency that may be beyond the typical background of patients or even therapists [12,13]. To fill these gaps, several studies have explored the feasibility of delivering rehabilitation treatments using low-cost, off-the-shelf gaming systems such as the Microsoft Kinect and PlayStation EyeToy [14-17]. Gaming controllers are intuitive to users, easy to repurpose, and more affordable, thereby offering a promising means for accessible home-based telerehabilitation. Gaming controllers can also measure motor performance objectively, toward remote assessment of patient status and progress by physicians [18].

As an example, the Novint Falcon can detect subtle differences in the kinematics of healthy and affected individuals [19] and evaluate patients’ motor learning as their rehabilitation progresses [20], through measurements of mean speed, peak speed, and path length traversed. These metrics have been previously used and upheld in robotic telerehabilitation of the upper limb to rapidly assess physical effort and movement accuracy and smoothness [21,22]. Other metrics can be used to assess patients’ motor performance, including range of motion [8,23], coordination [24,25], and amount of force exerted [14,26].

### Adherence to Rehabilitation Regimen

Although the mechanical framework of telerehabilitation has been successfully implemented in homes, patients often fail to comply with their home-based physical therapy, primarily because of the lack of motivation [27,28]. Acknowledging that sustained engagement is a prerequisite for successful outcomes, a large body of research has studied motivational interventions through game designs toward overcoming noncompliance in telerehabilitation [29-33]. For example, in an experimental study, Colombo et al [31] simulated a video game experience by displaying performance scores to improve motivation and adherence to the physical regimen. Similarly, in the study by Nijenhuis [29], a motivational messaging system was introduced to encourage future engagement in training sessions. Other studies have considered the use of serious games that do not aim primarily at entertainment for enhancing the physical rehabilitation experience [34-37]. For instance, Jonsdottir et al [38] have demonstrated the feasibility and efficacy of Rehab@Home, a therapeutic framework that simulates daily life activities in a virtual environment using Kinect. This gaming system was shown to increase gross motor function and improve patients’ experience and perception of health in patients with multiple sclerosis.

Overall, these studies have demonstrated that gamification increases engagement in rehabilitation exercises [37,39]. Yet, the full capacity of supplemental motivational interventions remains largely untapped as designers rarely emphasize the users’ intellect and interest to maintain prolonged engagement. Particularly in the context of rehabilitation, the age group of the majority of patients may not be conducive to the use of typical computer games that target young gamers [40,41]. Aiming to address the differential motivations of the elderly, Flores et al [27] pinpointed the gaming design criteria catering to the needs of both young and elderly users, which include (1) consideration of decreased sensorimotor abilities, (2) cognitively challenging elements, and (3) some degree of socialization.

### Citizen Science and Telerehabilitation

Following this line of work, we have previously demonstrated the potential utility of citizen science in increasing engagement and enjoyment in rehabilitation through the systematic interaction of environmental citizen science and robotics-based low-cost telerehabilitation technologies [19,42,43]. Citizen science projects address a wide range of scientific fields of inquiry. For example, on Stardust@Home, users can review images of an aerogel that was sent to outer space and flag traces of interstellar dust trapped in it [44]. In a different project, Foldit, volunteers fold virtual proteins and produce novel models of protein structures [45]. Citizen science could be a passive undertaking whereby citizen scientists lend computational power while their computers are idle [46,47]. The activities are not restricted to desktop computers and may also take place outdoors, where volunteers report of animal sightings or record air and water quality using their mobile phones [46,47].

In general, in citizen science projects, members of the public execute scientific tasks in authentic research projects, led by professional scientists and otherwise [46,47]. The contribution of volunteers typically involves data collection or data analysis and does not require specific expertise [47,48]. The motivation ascribed to citizen science projects is majorly intrinsic as participation is intellectually stimulating and promotes learning [48,49]. Moreover, the completion of individual tasks requires a small time commitment, allowing users to contribute at their own pace. Therefore, citizen science inherently satisfies the criteria identified by Flores et al [27], with the exception of social interaction, which must be separately addressed through new design interventions.

### Social Interactions as a Motivational Driver

Personal and social support, whether provided by practitioners, family, or friends, has been found to increase patients’ motivation toward performing exercise at home and to improve their mental well-being [50,51]. In the context of telerehabilitation, socially assistive robots, affording social interaction with patients while relaying treatments, were created [52]. The mere interaction with inanimate socially assistive robots was demonstrated to increase patients’ engagement in therapy and alleviate their feelings of stress and depression [52-54]. Building on this evidence, interhuman social interactions were introduced between the patient and their practitioner [52,55,56] and subsequently extended to include interactions with relatives and friends, and even strangers [30,52,57-59]. In all cases, patients expressed a strong preference to perform exercise with another person rather than alone and with a human partner rather than a virtual one [30,57,60]. Moreover, social interactions were demonstrated to improve motor performance [61]. Yet, the context in which social interactions were studied is largely limited to games.

Whether social interactions could benefit or hamper the success of citizen science–based rehabilitation treatments remains elusive. It is known that social presence alone should enhance user engagement and prolong participation in Web-based platforms through social comparison [62-65]. However, working in a team may also lead to the opposite outcome, whereby users could reduce their participation in an activity because of diffusion of responsibility, a sociopsychological phenomenon observed when an individual is less likely to assume responsibility of action in the presence of other individuals [66]. Diffusion of responsibility is moderated by several factors, including anonymity [67], group size [68], and division of labor [69], which can all be found in citizen science [70]. As a result, it is difficult to predict whether including social elements in citizen science–based rehabilitation could produce the sought motivational factor advocated in the study by Flores et al [27] or, instead, produce an adverse social phenomenon through diffusion of responsibility.

### Objectives

In this study, we sought to fill this gap in knowledge by examining the influence of computer-mediated cooperation on the engagement and motor performance of users involved in a rehabilitation exercise that integrated environmental citizen science and robotics-based technologies. We hypothesized that introducing cooperative tasks into citizen science would motivate users to extend their contribution by increasing the amount of scientific data they collect or analyze (productivity) and the time they spend performing the scientific task (persistence). This hypothesis rests on previous evidence that both of these measures are positively associated with motivation in goal-related activities [71]. In addition to increasing engagement, we expected that the integration of cooperation would improve users’ motor performance, reflected by their exertion of higher levels of physical effort. Finally, we hypothesized that the level of improvement in engagement and motor performance would be modulated by varying the degree of independence between paired users, whereby strengthening the interdependence between them would mitigate diffusion of responsibility.

To test our hypotheses, we created a novel, dedicated interface for Brooklyn Atlantis—a local citizen science project for environmental monitoring of the highly polluted Gowanus canal, located in Brooklyn, New York [72]. Our system enabled users to analyze pictures of the canal taken by an aquatic robot using a low-cost haptic controller, whose potential use in rehabilitation treatments on patients has been previously demonstrated by our group and other researchers [19]. Using the system, a pair of volunteers was presented with a list of descriptive keywords that may describe the objects in images. The volunteers sorted a list of labels together where one user allocates labels that describe objects in the image while the other discards irrelevant labels. Here, we report results for the effect of using the platform and the collaborative procedure on healthy people.

## Methods

### Hardware and Software

**Figure 1:**
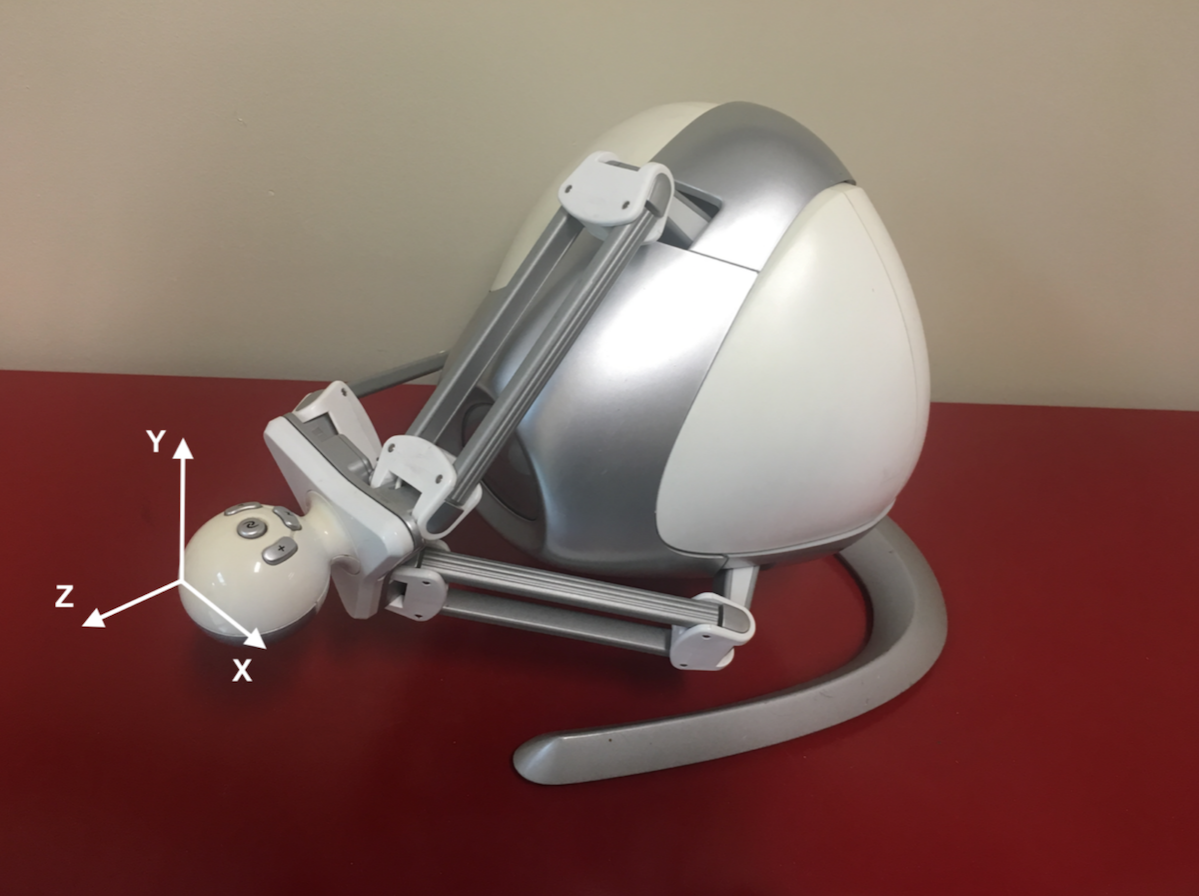
The Novint Falcon with the designated axes of motion.

**Figure 2:**
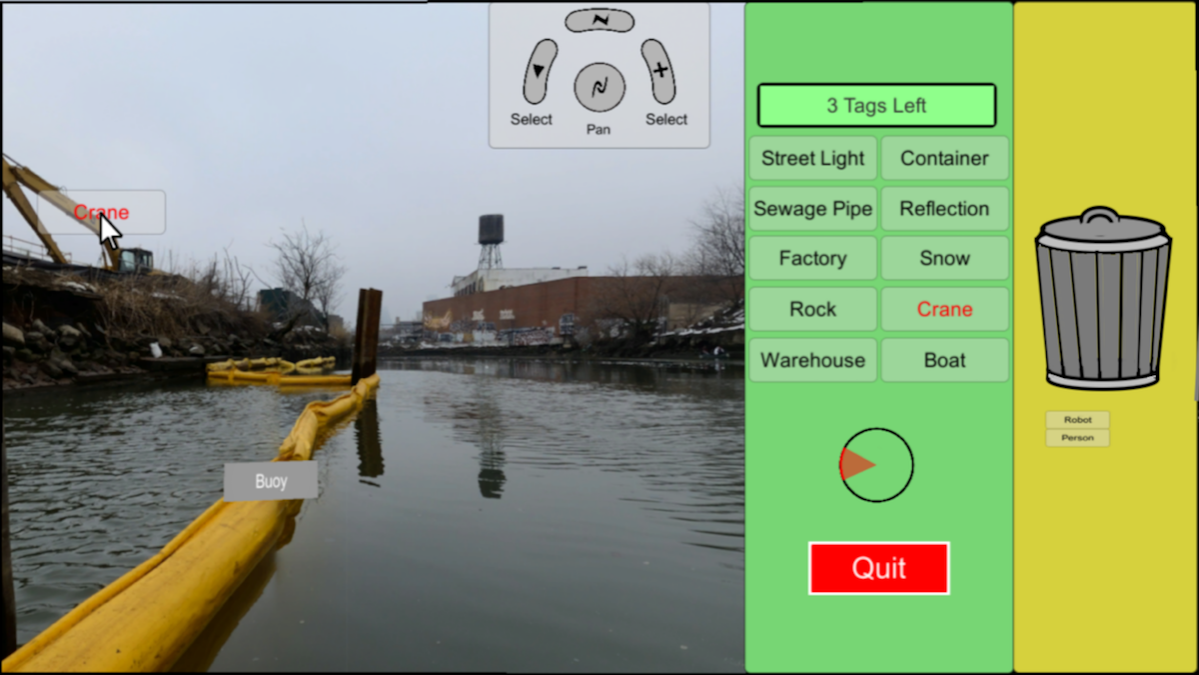
A screenshot of the user interface. On the left of the screenshot is a 360° image of the Gowanus canal. The user’s cursor is placing the label “Crane” onto the image, while a tag containing the word “Buoy” has already been placed. A reproduction of the Novint Falcon controller with a description of the function of each button is located on the upper right corner of the image. In the green panel, a counter of the number of labels that are yet to be assigned to the current image is displayed at the top. Below the counter, there is a list of 10 labels. The label “Crane” is highlighted in red as it is currently selected by the user. Below the list of labels is a visual feedback that represents deviation from the z-axis. A Quit button is situated at the bottom of the green panel. In the yellow panel, there is a garbage bin for eliminating labels that do not describe objects in the current image. The labels below it, “Robot” and “Person”, have been eliminated by the user.

All activities were performed using the Novint Falcon game controller, a low-cost haptic controller capable for use in 3 dimensions (Figure 1). The Novint Falcon offers translational hand movement with 3 degrees of freedom: left-right (x-axis), up-down (y-axis), and push-pull (z-axis). This device was demonstrated to provide effective fine-motor hand rehabilitation [73]. The system, developed using Unity 3D (Unity Technologies), displayed a 360° image of the Gowanus Canal on a computer screen (Figure 2). To explore the image, users pressed the middle button on the controller continuously and moved the controller in the general direction they wanted to rotate the view. A reproduction of the Novint Falcon interface was continuously shown on the screen, as a reference for the function of each button (Figure 2).

Movement was implemented in spherical coordinates, whereby motion of the controller along the x-axis (Figure 1) translated into azimuthal rotation (turning right or left in Figure 2) and motion of the controller along the y-axis (Figure 1) translated into elevational rotation (turning up or down in Figure 2). As the motion along the push-pull axis did not convey a meaningful function (zoom was not offered), a highly resistive force was applied in this direction to prevent motion. In addition, visual feedback was added to the system, such that the image of the Gowanus Canal would fade in response to motion along the z-axis, either pushing or pulling. The deviation from the z-axis was further conveyed through a black circle and radiating cone, portraying where the user is facing and how far off the axis they are located (Figure 2).

**Figure 3:**
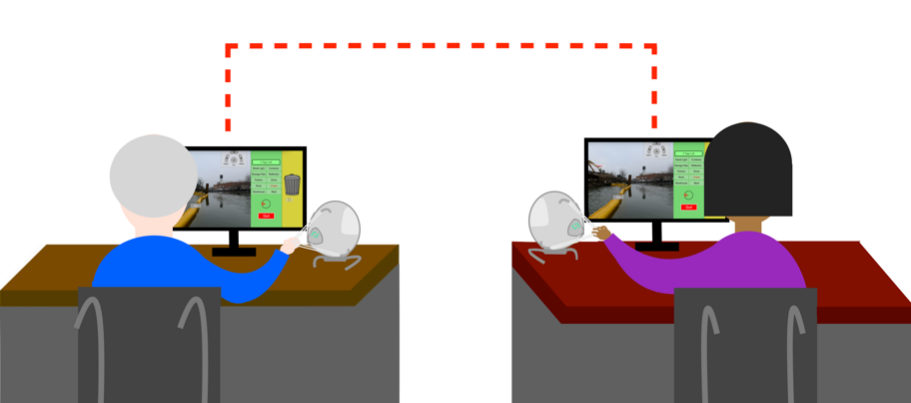
Schematic of two cooperating users classifying images remotely from two different computers in separate rooms.

As part of the citizen science image classification project, 2 tasks were implemented in the system. The first task consisted of tagging objects observed in the images using labels from a list, located on the right of the 360° image (green panel in Figure 2). The second task entailed eliminating labels from the list that were not in the image by allocating them to the trash bin, located on the right of the list of labels (yellow panel in Figure 2). When 2 users performed the tasks together, each user had independent control over exploration of the 360^°^ image. Social cues were conveyed through the system as cooperating users could view their partner’s actions in real time. That is, the user assigned with the tagging task could see a list of the eliminated labels forming below the trash bin (Figure 2). Similarly, the user assigned with the trashing task could see the labels assigned to the image (Figure 2). The presence of a peer was further made evident by highlighting a label in red, indicating that it was selected by the peer. An illustration of the setup is depicted in Figure 3.

Users were able to select labels by pressing the right or left button on the controller, depending on their dexterity. Once a button was pressed, the label was tethered to the cursor and effectively dragged by it. To deselect a label, or to release it at a desired location, users pressed the controller button again. Once assigned, labels were replaced by others from a predetermined sequence of 49 labels, all of which were previously contributed by citizen scientists in Brooklyn Atlantis in our previous research [19,74-77]. To maintain fluidity of the image classification process and avoid oversaturation of images with tags, an image was replaced by another image after 5 tags were assigned. A tag counter was displayed on the screen to inform the user of the number of tags assigned to the current image (Figure 2).

Users were able to terminate the activity by pressing the red *Quit* button on the screen (Figure 2). To simulate the different levels of interdependence in the cooperation between individuals, 2 types of termination were considered in the experiments: independent termination (IT), whereby users could continue contributing to the project even after their peer had quit, and joint termination (JT) whereby termination by 1 user ceased the session for the other user too.

### Experimental Procedure

This study was carried out in compliance with the institutional review board (IRB) at New York University (IRB FY2016-184). Overall, 120 members of the university community, with a mean age of 27.36 (SD 8.28) years, were recruited and subjected to one of 3 conditions (Table 1): IT (50 volunteers), JT (48 volunteers), and control (22 volunteers). Although control subjects performed both tagging and trashing, volunteers in IT and JT cooperatively carried out the activity such that half of the volunteers (25 and 24 volunteers in IT and JT, respectively) performed only tagging and the other half (25 and 24 volunteers in IT and JT, respectively) performed only trashing. In the control condition, the volunteer was able to withdraw from the activity at any time.

**Table 1:** A summary of the experimental conditions tested.

Condition and task assignment		Cooperation	Number of volunteers
**Control**			
	Tagging and trashing	Absent	22
**Independent termination**			
	Tagging	Present	25
	Trashing	Present	25
**Joint termination**			
	Tagging	Present	24
	Trashing	Present	24

Recruitment and experimental procedures were standardized through scripts and a PowerPoint presentation. We recruited volunteers in public spaces on campus. During recruitment, we verbally introduced potential participants on campus to the notion of citizen science following a script. Once recruited, paired volunteers were brought into 2 separate private rooms to simulate Web-based cooperation as envisioned in future application within robotics-based telerehabilitation. They did not know who their peer was.

All participants were subjected to the same experimental protocol. Before beginning the experiment, participants were given an overview of the Gowanus Canal and Brooklyn Atlantis using a PowerPoint presentation. Through the presentation, within cooperative conditions, IT and JT, participants were notified that they will be working together with a peer and were instructed to complete their assigned task only. They were informed that they may withdraw at any point they would like and whether their withdrawal will terminate their peer’s participation (JT) or not (IT). Upon signing a consent form, the participants underwent a tutorial teaching them how to use the Novint Falcon and the system. After the tutorial, they were connected with their peer and began carrying out their tasks. Users who were subjected to the control condition carried out both tasks, tagging and trashing. Users who were subjected to cooperative conditions were randomly assigned to one of the 2 tasks. They performed the exercise until they pressed the quit button. After quitting, the participants rated their experience on a 7-point Likert scale in response to the statements “I enjoyed this activity” and “This activity was fun.” Once the volunteers submitted their answers, the experiment was concluded.

### Data Collection and Analysis

#### Data Acquisition

For each user, 3 datasets were created. The first dataset documented information on tag allocation, including tag content, time of allocation, and allocating user identity number. The second dataset recorded users’ scores of enjoyment. The third dataset consisted of the Novint Falcon controller position in 3D space, recorded at a sampling rate of 60 positions per second. The collected data were used to quantify user engagement, enjoyment, and motor performance.

#### Data Processing

User engagement was evaluated through their productivity and persistence [71]. Productivity was measured as the number of labels processed by the user. Persistence was measured as the time spent performing the activity [71]. Users’ enjoyment was evaluated from surveys. Interrater reliability was validated using the Cronbach alpha [78]. Enjoyment was scored by averaging the ratings on the multiple questions for each user, linearly scaling between 0 (Likert scale 1) and 1 (Likert scale 7), and normalizing using an arcsine transformation by considering the proportional nature of the variable [79].

The trajectory of the controller was examined from the recording of consecutive points in space over time. A total of 3 motion metrics were evaluated from the trajectory, namely, the controller’s mean speed, peak speed, and path length. For each trial, the instantaneous speed was estimated using a backward Euler scheme on the sampled positions from the haptic device. The mean speed was computed by averaging instantaneous values over the whole trajectory and the peak speed as the maximum value among the 90th percentile from the trajectory [80]. The path length was measured as the sum of distances between pairs of consecutive data points.

#### Statistical Analysis

The influence of cooperation on engagement, enjoyment, and motor performance was investigated by fitting each variable into a generalized linear-mixed effects model [81], specifying condition (3 levels: control, IT, and JT) as an independent variable and both pair identity and task assignment (tagging and trashing) as random effects (R *lme4* package version 1.1-15 [82]). To improve the normality of the model residual, we specified a Gaussian family with a log link for persistence and enjoyment, a Poisson family with a log link for productivity, and a gamma family with a log link for motor performance. The significance of the influence of conditions was tested using a likelihood ratio test, comparing the model against a null model in the absence of the condition as the independent variable. When significant effect was found, post hoc analysis was performed using the Dunnett test (R *multcomp* package version 1.4-8 [83]).

Next, we evaluated the influence of the modality through which cooperation was implemented on engagement, enjoyment, and motor performance. Specifically, we fitted each variable into a generalized linear mixed-effects model, specifying condition (2 levels: IT and JT), task assignment (2 levels: tagging and trashing), and the interaction between them as independent variables, and pair identity as a random effect. The same error family as the previous model was used for the corresponding variable. To test the significance of the interaction term, the full model was tested against a null model without the interaction using a likelihood ratio test. In case a significant interaction was found, the difference between tasks was further examined within each condition, by specifying task as an independent variable and pair identity as a random effect. In case the interaction was not significant, we removed the interaction from the full model, and the effects of condition and task were tested using a likelihood ratio test, individually, comparing against a null model.

Although not part of our original hypotheses, we also tested the influence of social presence on individual speed performance. In each pair of cooperating peers in IT, the trajectory of the more persistence was partitioned into 2 parts, before and after their peer had quit. Users’ mean and peak speeds were fitted into separate generalized linear mixed-effects models, specifying the time partition (2 levels: before and after) as an independent variable and user identity as a random effect. A gamma family with log link was specified to normalize the model residual. Users’ speeds before peer withdrawal were compared with their speeds following peer withdrawal using a likelihood ratio test, comparing the model against a null model in the absence of the time partition as the independent variable.

For all statistical tests, we set the level of significance at alpha=.05.

## Results

### Influence of Cooperation on Engagement

**Figure 4:**
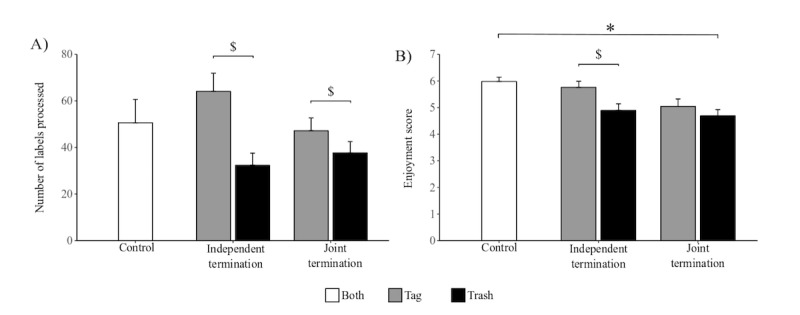
Engagement of users in the activity. A) number of labels processed by participants in each condition. B) rate of enjoyment for each condition. The vertical lines represent standard errors. *: statistically different means among conditions. $: statistically different means among tasks.

On average, users processed (tagged or trashed) a mean of 46.35 (SD 3.16) labels, spending 16.37 (SD 0.63) min. Neither productivity nor persistence were found to differ among conditions (*χ*^*2*^_2_=0.1 *P*=.92 and *χ*^*2*^_2_<0.1; *P*=.79, respectively; Figure 4). However, the level of enjoyment was found to vary among conditions (*χ*^*2*^_2_=10.5; *P*=.005; Figure 4), with JT users rating the activity significantly lower than control users (z=3.25; *P*=.002). By contrast, IT users did not rate the activity significantly different from control users (z=1.94; *P*=.08).

### Influence of Cooperation on Motor Performance

**Figure 5:**
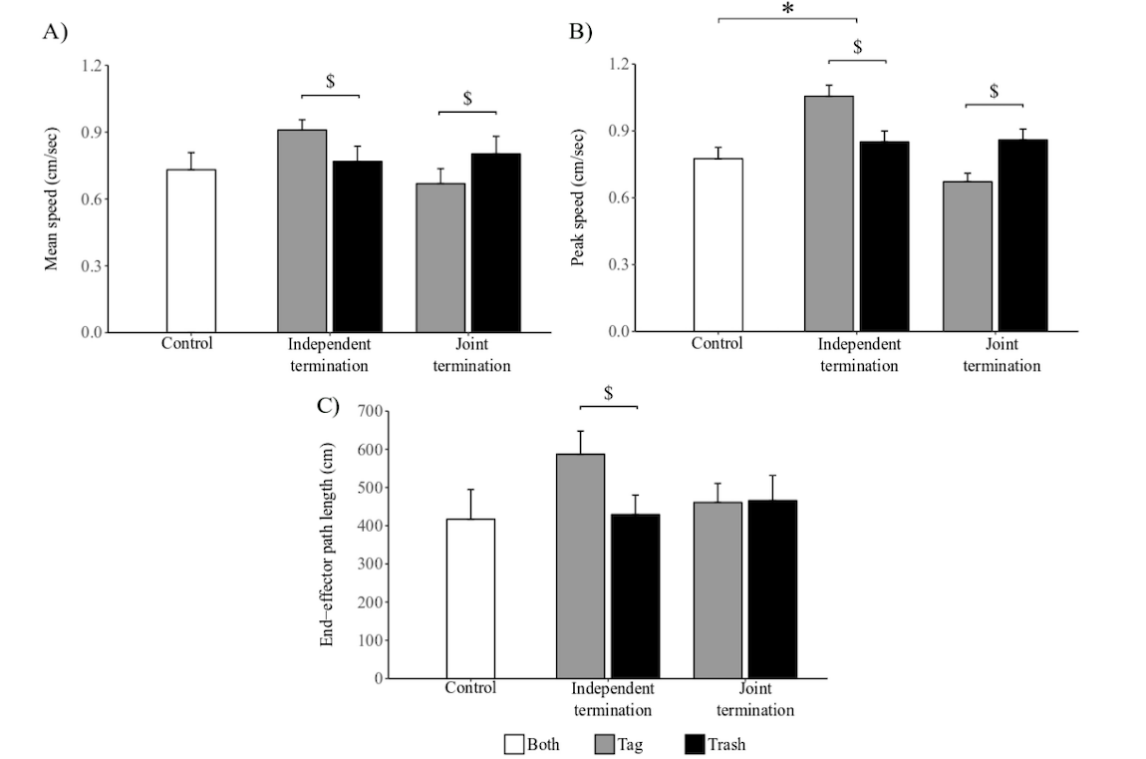
Motor metrics. A) mean speed in each condition, B) peak speed for each condition, C) path length traversed by the controller in each conditions. The vertical lines represent standard errors. * represents statistically different means among conditions. $ represents statistically different means among tasks.

With regard to motor performance, the mean speed did not differ among conditions (*χ*^*2*^_2_=2.6; *P*=.26). Contrarily, we determined a significant variation of peak speed among conditions (*χ*^*2*^_2_=7.7; *P*=.02; Figure 5). Although post hoc comparisons failed to identify a significant difference between JT and control conditions (z=0.11; *P*=.99), we registered a significant difference between IT users and control users (z=2.44; *P*=.02). The path length was not significantly different among conditions (*χ*^*2*^_2_=4.1; *P*=.12).

### Influence of Modality on Engagement

Testing for the influence of cooperation modality on productivity, we found a significant interaction between condition and task (*χ*^*2*^_2_=43.1; *P*<.001). When investigating the effect of task assignment in each condition, we found a significant difference in productivity between the tasks in both conditions, IT (*χ*^*2*^_1_=265.7; *P*<.001) and JT (*χ*^*2*^_1_=25.5; *P*<.001). We failed to identify a significant interaction between condition and task with regard to persistence (*χ*^*2*^_1_=3.3; *P*=.06). An interaction between condition and task was not found in enjoyment as well (*χ*^*2*^_1_=1.1; *P*=.29). Enjoyment was significantly different between the tasks (*χ*^*2*^_1_=7.7; *P*=.005), whereas condition failed to reach significance (*χ*^*2*^_1_=2.8; *P*=.09).

### Influence of Modality on Motor Performance

With regard to the performance metrics, a significant interaction between condition and task was found to influence path length (*χ*^*2*^_1_=6.3; *P*=.01), mean speed (*χ*^*2*^_1_=7.4; *P*=.006), and peak speed (*χ*^*2*^_1_=25.8; *P*<.001; Figure 5). For path length, we found a significant difference between task assignment in IT condition (*χ*^*2*^_1_=11.3; *P*<.001), whereas we did not find one in JT condition (*χ*^*2*^_1_<0.1; *P*=.90). For mean speed, a significant difference between tasks was found in IT condition (*χ*^*2*^_1_=6.2; *P*=.01) but not in JT condition (*χ*^*2*^_1_=2.4; *P*=.12). Finally, for peak speed, a significant difference was found between tasks in both IT and JT conditions (*χ*^*2*^_1_=16.1; *P*<.001 and *χ*^*2*^_1_=11.0; *P*<.001, respectively).

**Figure 6:**
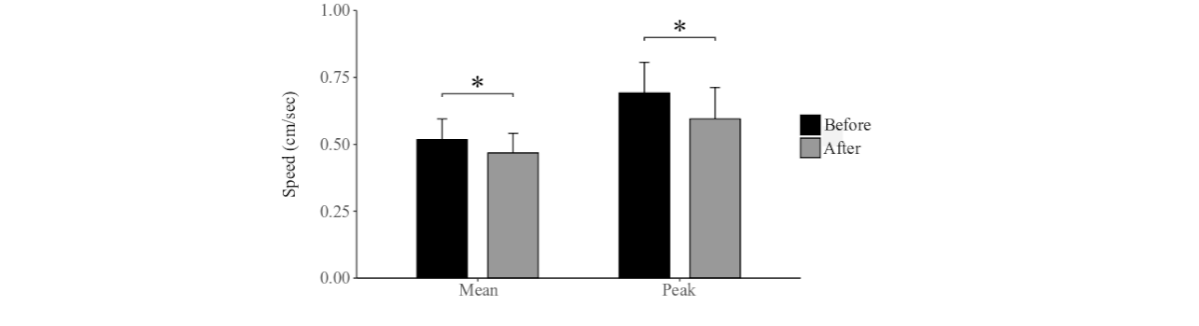
Differences in mean and peak speeds of the more persistent users in condition IT, before and after their peer has withdrawn. The vertical lines represent standard errors. * represents statistically different means among conditions.

In IT, volunteers significantly reduced their mean and peak speeds following their peer’s quitting (z=2.97; *P*=.002 and z=3.30; *P*<.001, respectively; Figure 6).

## Discussion

### Principal Findings

Citizen science is an effective means for improving rehabilitation treatments. Patients undergoing physical rehabilitation have shown a strong preference toward exercise embedded with citizen science and were more likely to repeat it at the cost of their time commitment [19]. Although social interactions hold potential to further increase patients’ engagement in rehabilitation [30,57], the modality in which they are framed could widely shape the outcomes of the treatment [84]. In this study, we attempted to elucidate the influence of computer-mediated cooperation on motor performance during a citizen science activity, mediated by a low-cost haptic device.

We designed a series of experiments simulating an authentic telerehabilitation setting, where participants remotely cooperated in the analysis of environmental images using low-cost haptic devices. From survey instruments and direct measurements of motor activities, we sought to quantify the potential effect of cooperation in telerehabilitation. Our results indicate that 2 elements, interdependence and task assignment, can influence the effects of cooperation on engagement and motor performance, thereby offering a potential means for improving rehabilitation treatments. However, the way in which these 2 variables interact to influence the response of the subjects may challenge one’s intuition.

In partial contrast with our hypotheses, we did not find that cooperative division of labor, such that each user is assigned to a different task toward a shared goal [85], is always conducive to higher engagement. Although we did not register a difference in the level of engagement between control users and users who cooperated via IT, we found an expected reduction in enjoyment for users who cooperated via JT. In our experimental design, JT was implemented to promote interdependence between users, which we had initially identified as a key factor to mitigate diffusion of responsibility and coerce users to persist for a longer period of time [86,87]. However, it is likely that JT was accompanied by other confounding factors, contributing to a reduction in enjoyment.

It is tenable that we inadvertently introduced random termination in the trials where users faced uncertainty with regard to the timing of termination, as the other player could terminate the task at any time. This uncertainty about the horizon of the relationship with the other player was shown in previous research to impact the degree of cooperativeness of players negatively [88,89], where the more termination becomes likely, the less cooperation has been observed [90]. In game theory, random termination has been shown to discount the payoff of players’ actions [88,89] such that players would try to avoid losses and become less cooperative. Such weakening of cooperation also results in lower levels of enjoyment and satisfaction [91]. Similar dynamics likely emerged in the proposed citizen science–based telerehabilitation activity, thereby calling for future research to explore alternative strategies that could promote interdependence between users. For instance, we could attempt at a priori identifying a predetermined length for the trials, by matching patients undergoing a similar rehabilitation therapy.

Our findings also provided insight into the role of social interactions on motor performance. Measuring relevant kinematic variables is central to the notion of telerehabilitation, whereby supplying care providers with clinical information will enable them to track patients’ status and adjust their rehabilitation program remotely and efficiently [18]. Ideally, care providers could also infer abnormal, compensatory movement from the data and instruct patients to correct it [18]. Mean speed, peak speed, and path length traversed [19,92] have been used in robotic telerehabilitation of the upper limb as indicators of motion quality [14,21,22]. In addition to the evaluation of motor performance, the physical effort exerted by an individual can also be linked to their motivation to perform the exercise task. Investing greater effort to complete a challenging task often leads to self-determined behavior, resulting in a sense of competence [93] and an increase in intrinsic motivation [94].

Although we expected that users would improve their motor performance because of cooperation, we found a modest reduction in motor performance similar to the discussed reduction in enjoyment. More specifically, motor performance of users cooperating via JT was similar to control with regard for all the selected metrics, whereas IT resulted in higher values of the peak speed relative to control. It is possible that random termination could explain the observed difference, where a user would not invest the same effort when faced with the potential that his/her work could be vanished because of exogenous termination of the activity by the peer. Future research should seek to explore alternative modalities to favor cooperation, without challenging enjoyment and effort that are key to the success of rehabilitation. The notion of setting intervals for the exercises could be a viable approach, whereby it could mitigate the harmful effects of random termination, while leveraging the beneficial role of cooperation. In fact, analyzing time variations of motor performance of users who cooperated via IT, we discovered that the speed of the more persistent users in IT significantly reduced following their peer’s withdrawal. This confirms the intuition that social interaction should prompt individuals to exert more effort in their task, thereby calling for future studies to engineer social interactions toward improving recovery and patients’ self-perception of physical capacity [95,96].

In addition to the type of termination, we found that task assignment can modulate user engagement. We found that cooperating users in IT condition assigned with tagging were more engaged than their peers who were assigned with trashing. The difference in engagement may be attributed to the perceived nature of the task a user has been assigned to. In fact, engagement is positively associated with the identifiability of the share an individual contributes to the groupwork [87,97], that is, individuals whose contribution is more valued and recognized by group peers are more likely to be motivated to perform their task. Conversely, individuals whose contribution is less important and recognized by others in the group are less motivated to perform their task. Therefore, the dissimilarity between tagging and trashing tasks could lead to unequal levels of engagement, with tagging users being more engaged than trashing users. This observation calls for further studies in which targeted design interventions will be explored to investigate differences between cooperation and collaboration, where individuals are assigned to the same task. It is tenable that cooperating individuals should perform better when assigned with a common group task rather than interdependent tasks [84]. Working in a collaborative setting where they fulfill identical functions jointly in support of a shared goal [85] could bolster team cohesion and lead to even higher performance and satisfaction among users [98].

Evidence shows that group cohesiveness can be improved with increasingly overt sharing of information, leading to greater engagement in group tasks [99]. Recently, we showed that the mere presentation of *social foot prints*, digital cues that suggest the presence of other Web users can be used to increase the amount and duration of physical exercise during citizen science activities [64]. In a different study, using a virtual peer operating in open- and closed-loop paradigms, we demonstrated that bidirectional flow of social information can substantially increase user contribution to a citizen science project [75]. Seemingly, as more social presence is conveyed between Web users, an increasingly trusting climate is created, conducive to cooperation [100]. In future studies, one could explore how sharing of personal information such as age, location, and interests can impact trust and engagement in Web-based citizen science telerehabilitation [101].

Citizen science contributes to a sense of community in cooperative telerehabilitation through the introduction of scientific content. Unlike gaming-based motivational interventions, this study capitalizes on human intellect as an intrinsic motivator. Previously, we had shown that users prefer to perform an exercise associated with scientific content [19,92]. Beyond the intellectual stimulus, citizen science adds virtue and a sense of contribution to the activity, which is not found in the majority of serious games. Web-based social platforms where individuals share personal values often create communities with which contributors can identify [48]. In telerehabilitation in particular, patients can benefit from such an environment that could alleviate the isolation many of them experience [102,103].

### Limitations

Although our work brings forward evidence in favor of the use cooperative citizen science in rehabilitation, it comes with a number of limitations. First, the difference in engagement that we observed among conditions was moderate. It is possible that because citizen science is inherently engaging [92], additional motivational interventions such as social interactions offer only a weak enhancement to engagement, limited by a ceiling effect. To surpass such a ceiling effect, it would be beneficial to explore the role of cooperation in citizen science–based rehabilitation in a longitudinal study, where persistence is measured by the frequency patients choose to engage in exercise and productivity is measured as aggregated contribution.

Second, we studied motor performance using the Novint Falcon, a haptic device which is no longer being produced. However, the fine-motor tasks imparted by the Novint Falcon can be achieved using other haptic devices. For instance, surgical delta robots such as the Force Dimension and Phantom offer movement with 6 degrees of freedom and can apply a comparable amount of force feedback [104-106].

Third, in this study, we recruited healthy subjects from New York University campuses. Our findings may be narrowly generalizable as the sample consists of healthy individuals from the Brooklyn area with access to high education. Although we drew our sample from different programs of the university, the volunteers may have distinctly different interests and motivations from the typical patient undergoing rehabilitation. For example, the participants in this study may have greater interest in science or in restoration of the Gowanus Canal than the average person. Offering a wider range of citizen science projects to choose from based on personal interests might further improve enjoyment, engagement, and motor performance. Future research with patients from diverse backgrounds in a clinical setting will elucidate effects of this work on clinical outcomes.

### Conclusions

We offer evidence for the utility of cooperation in improving engagement in citizen science–based telerehabilitation. Citizen science can offer intellectual stimulus and a community for patients to engage with and relate to. It attends the needs of more patients, including those who are less interested in traditional gaming [27,37], thereby extending the benefits of adherence to home-based physical therapy to a larger population. The value of this study can be expanded to other domains that rely on user participation and engagement, including Web-based consumer platforms [107], social networks [108], crowdsourcing efforts [109], and general game design [91]. Ultimately, we anticipate our approach will be translated into low-cost technology for telerehabilitation and help patients reach their full potential recovery.

## Abbreviations

IRB: institutional review board
IT: independent termination
JT: joint termination
